# Treatment outcome of thalidomide based regimens in newly diagnosed and relapsed/refractory non-transplant multiple myeloma patients: a single center experience from Thailand

**DOI:** 10.1186/1756-8722-3-1

**Published:** 2010-01-05

**Authors:** Pimjai Niparuck, Ladda Sorakhunpipitkul, Vichai Atichartakarn, Suporn Chuncharunee, Artit Ungkanont, Pantep Aungchaisuksiri, Teeraya Puavilai, Saengsuree Jootar

**Affiliations:** 1 Division of hematology, Department of Medicine, Ramathibodi Hospital, Mahidol University, Thailand

## Abstract

**Background:**

Thalidomide based regimen is an effective and well tolerated therapy in multiple myeloma (MM) patients, however, there were a small number of studies written about the results of thalidomide therapy in non-transplant MM patients. We therefore conducted a retrospective study of 42 consecutive patients with newly diagnosed and relapsed/refractory MM treated with thalidomide- based induction regimens followed by thalidomide maintenance therapy.

**Results:**

Induction regimens with thalidomide and dexamethasone, and the oral combination of melphalan, prednisolone and thalidomide were administrated in 22 and 16 patients, respectively. The remaining 4 patients received other thalidomide- containing regimens. Twenty-nine patients received thalidomide as a salvage regimen. Twenty-three out of 26 patients achieving complete remission (CR) and very good partial remission (VGPR) received thalidomide maintenance. Of the 41 evaluable patients, median time of treatment was 21 months (3- 45 months), ORR was 92.7% with a 63.4% CR/VGPR. With a median follow up of 23 months, 3-year- PFS and 3-year-OS were 58.6 and 72.6%, respectively. Median time to progression was 42 months. While 3-year-PFS and 3-year-OS in non-transplant patients receiving thalidomide maintenance therapy were 67 and 80%, respectively.

**Conclusions:**

Prolonged thalidomide therapy enhanced survival rate and less frequently developed serious toxicity in non-transplant multiple myeloma patients.

## 

To the editor:

Thalidomide based therapy for multiple myeloma (MM) improves the response and the complete remission (CR) rates in previously untreated and relapsed/refractory MM (overall response rate was 48- 73% with a 5- 10% CR) [1,2]. In this study, we performed a retrospective study of 42 newly diagnosed and relapsed/refractory MM patients treated with thalidomide based regimens without upfront ASCT at Ramathibodi Hospital during January 2005-October 2008. Thirteen and 29 patients were previously untreated and relapsed/refractory MM, respectively (Table 1). Twenty-two patients received thalidomide 200 mg/day and oral dexamethasone 20- 40 mg/day (d1-4) every 2 weeks, 16 patients received oral melphalan 4 mg/m^2^/day (d1-7), prednisolone 40 mg/m^2^/day (d1-7) and thalidomide 100 mg/day every 4 weeks, 3 patients received thalidomide 200-400 mg/day and the remaining 1 patient received thalidomide 100 mg/day, pegylated liposomal doxorubicin i.v. 40 mg/m^2^/day (d1) and oral dexamethasone 40 mg/day (d1-4, 9-12) every 4 weeks. Eighty-eight percents (23/26 patients) achieving CR/VGPR (very good partial remission) received thalidomide maintenance therapy (100-200 mg/day). Aspirin 65- 325 mg/day or warfarin 1.5 mg/day was given to all patients for deep vein thrombosis prophylaxis. Of the 41 evaluable patients, median treatment period was 21 months (3- 45 m). The ORR (overall response rate) was 92.7%, with a 63.4% CR/VGPR. Median number of courses to achieve PR and CR/VGPR were 4 (range, 2-13) and 6 courses (range, 2-16), respectively. There was no difference in ORR and CR between frontline and salvage therapy groups (92.3% vs 93%) and (39% vs 23%), respectively. The ORR and CR rate for those treated with thal/dex were slightly higher than those treated with MPT (95.2% vs 87.5% and 38% vs 25%). Median follow up was 23 months, 3-year-OS and 3-year-PFS were 72.6 and 58.6%, respectively. Median TTP was 42 months, non- VGPR/CR patients had significant poorer PFS by multivariate analysis (*p *= 0.01) and non-responders had significant shorter OS (*p *= 0.01). In maintenance group, median treatment duration was 14 months (4-37 m). Three-year-PFS and 3-year-OS were 67 and 80%, respectively. Toxicities were constipation (81%), neuropathy (67%), muscle weakness in the legs (5%), infection (7%) and thrombosis (5%). New agents for treatment of MM with no planned ASCT show the CR/VGPR rates of 50- 80% with a PFS of 2 years [3-5]. The CR/VGPR rates in our patients were also high that might be associated with a prolonged use of thalidomide induction. Thalidomide maintenance in CR/VGPR patients provided impressive survival benefit. Hence, thalidomide is an effective therapy for MM and prolonged thalidomide use had the survival benefit and had minimal serious toxicity in non-transplant MM patients. To date, MM remains incurable. Novel agents continue to be developed and are eagerly awaited [5-7].

**Table 1:** Patients' characteristics and treatment outcomes of previously untreated and relapsed/refractory multiple myeloma

		ORR		CR/VGPR		PFS			OS		
		
Characteristics	Total patients (N = 42)	No. of Patients %	p-value	No. of patients	p-value	HR	95% CI	p-value	HR	95% CI	p-value
Age (years), median (range)	62,(36-75)										
≤ 60	17	15(94)	0.83	9(56.3)	0.45	2.95	0.98-8.81	0.05	0.81	0.09-7.27	0.85
> 60	25	23(92)		17(68)							

Sex											
Male	21	18(85.7)	0.79	13(65)	0.91	0.77	0.25-2.38	0.65	2.06	0.34-12.68	0.44
Female	21	20(95.2)		14(66.7)							

Prior treatment											
Yes	29	26(92.6)	0.95	19(67.9)	0.69	3.68	0.91-10.28	0.06	0.87	0.96-7.88	0.9
No	13	12(92.3)		8(61.5)							

International staging system											
I, II	8, 18	24(92.3)	0.97	15(57.7)	0.93	6.30	0.73-54.01	0.09	2.22	0.20-24.57	0.51
III	13	12(92.3)		8(61.5)							
No data	3										

M-protein subtype											
IgG, IgA, IgM	23, 8, 1	27(87.1)	0.32	19(61.3)	0.86	3.19	0.64-15.91	0.16	1.21	0.13-11.65	0.87
Kappa, Lamda	3, 6	9(100)		6(66.7)							
Unknown type	1										

Serum creatinine level											
< 2 mg/dl	34	31(91.2)	0.43	22(64.7)	0.57	0.74	0.08-6.72	0.79	0.03	0.01-856.9	0.50
≥ 2 mg/dl	8	7(100)		4(57.2)							

Serum β2 M level, μg/ml											
≤ 5	26	24(92.3)	0.53	17(65.4)	0.79	4.89	0.55-43.88	0.16	1.97	0.18-21.81	0.58
> 5	13	11(84.6)		8(61.5)							
No data	3										

Response to treatment											
Yes	38	-	-	-	-	0.15	0.04-0.61	0.01	0.03	0.01-0.35	0.01
No	3										

CR/VGPR											
Yes	26	-	-	-	-	0.14	0.04-0.47	0.01	0.21	0.03-1.48	0.12
No	15										
